# Associations among osteocalcin, leptin and metabolic health in children ages 9–13 years in the United States

**DOI:** 10.1186/s12986-017-0171-9

**Published:** 2017-03-07

**Authors:** Kelly Virecoulon Giudici, Joseph M. Kindler, Berdine R. Martin, Emma M. Laing, George P. McCabe, Linda D. McCabe, Dorothy B. Hausman, Lígia Araújo Martini, Richard D. Lewis, Connie M. Weaver, Munro Peacock, Kathleen M. Hill Gallant

**Affiliations:** 1 Department of Nutrition, School of Public Health, University of São Paulo, Avenida Doutor Arnaldo 715, São Paulo, CEP 01246-904 Brazil; 2 Department of Foods and Nutrition, University of Georgia, 305 Sanford Dr, Athens, GA 30602 USA; 3 Department of Nutrition Science, Purdue University, 700 W. State Street, West Lafayette, IN 47907 USA; 4 Department of Statistics, Purdue University, 150 N. University Street, West Lafayette, IN 47907 USA; 5 Department of Medicine, Indiana University School of Medicine, 545 Barnhill Dr, Indianapolis, IN 46202 USA

**Keywords:** Osteocalcin, Children, Leptin, Obesity, Glucose, Insulin

## Abstract

**Background:**

This study aimed to investigate the relationships among osteocalcin, leptin and metabolic health outcomes in children ages 9–13 years.

**Methods:**

This was a cross-sectional analysis of baseline data from 161 boys and 157 girls (ages 9–13 years) who previously participated in a double-blinded randomized placebo controlled trial of vitamin D supplementation. Relationships among fasting serum total osteocalcin (tOC), undercarboxylated osteocalcin (ucOC), leptin, and metabolic health outcomes were analyzed.

**Results:**

Approximately 52% of study participants were obese based on percent body fat cutoffs (>25% for boys and >32% for girls) and about 5% had fasting serum glucose within the prediabetic range (i.e. 100 to 125 mg/dL). Serum tOC was not correlated with leptin, glucose, insulin, HOMA-IR, or HOMA-β after adjusting for percent body fat. However, serum ucOC negatively correlated with leptin (partial r = −0.16; *p* = 0.04) and glucose (partial r = −0.16; *p* = 0.04) after adjustment for percent body fat. Leptin was a positive predictor of insulin, glucose, HOMA-IR, and HOMA-β after adjusting for age, sex and percent body fat (all *p* < 0.001).

**Conclusions:**

These data depict an inverse relationship between leptin and various metabolic health outcomes in children. However, the notion that tOC or ucOC link fat with energy metabolism in healthy children was not supported.

**Clinical trial registration number:**

NCT00931580.

## Background

The relationships between bone, adiposity and energy metabolism have been of recent interest [1–7]. Whereas excess adiposity may benefit bone development as a result of earlier and prolonged maturation [8], some studies have identified fat mass as a negative determinant of skeletal endpoints after adjusting for pertinent confounders [8, 9]. One explanation for a potentially adverse influence of adiposity on bone involves obesity-related metabolic health outcomes, specifically insulin resistance [10]. This is of particular importance considering that obese children are overrepresented in pediatric skeletal fracture cases [11], and that certain bone-derived factors are understood to contribute to energy metabolism [12]. Therefore, elucidating the mechanisms by which adiposity influences bone, as well as how bone-derived factors influence the fat-bone relationship and metabolic health, warrants further investigation.

Leptin is a hormone that has received considerable attention in relation to the fat-bone connection, despite its better-known role in appetite regulation [1]. Leptin has been shown to influence bone through numerous mechanisms [1], thus it is not surprising that the role of leptin on bone is both complex and controversial [13]. Ducy et al. [1] identified leptin as a potent inhibitor of bone formation acting through a central nervous system mechanism. In mice, intracerebroventricular leptin infusion stimulated hypothalamic leptin receptor (LEPR) expression, promoting norepinephrine release, and in turn, activating osteoblast β2-adrenergic receptors. Ultimately, this resulted in altered bone metabolism in favor of bone resorption [1, 14]. Conversely, peripheral leptin signaling through LEPR in bone marrow stromal cells promoted differentiation to the osteoblastic lineage over the adipocytic lineage [15]. Further, LEPR signaling in osteoblasts increased osteoprotegerin (OPG) and decreased the receptor activator of nuclear factor κB ligand (RANKL), resulting in decreased osteoclastogenesis. Thus, in contrast to the bone-resorptive effects of leptin via the central nervous system, peripheral leptin signaling at the level of the skeleton appears to favor bone formation [13].

The bone-derived osteocalcin, a protein produced by osteoblasts, is also suspected to play an integral role in the link between fat, bone, and metabolic health [16, 17]. However, there is still considerable debate surrounding the role of osteocalcin, including its sub-fractions (i.e., carboxylated [cOC] and undercarboxylated [ucOC]), in the complex fat-bone connection. This is especially true for pediatric populations. Osteocalcin-null mice versus wild-type littermates have greater visceral fat, and consequently, present with hyperglycemia and are insulin resistant [3]. In humans, there is conflicting evidence for a relationship between tOC and glucose metabolism with some reports showing no relationship [18, 19], and others showing an inverse relationship between tOC and glucose concentrations [20–22]. In contrast, ucOC seems to play a role in pancreatic β-cell proliferation and insulin secretion, as well as improving peripheral insulin sensitivity through the regulation of the adipokine adiponectin [3, 4]. Whereas experimental [3, 4] studies have suggested that ucOC might play a more pivotal role in energy homeostasis than tOC, obese versus normal weight children have lower tOC, which is a negative predictor of serum leptin and insulin resistance [23], thus the relative importance of tOC and ucOC on metabolic health is not currently well defined. Additionally, the effects of physical activity through skeletal loading on the bone-fat-energy metabolism relationship have been described, with ucOC proposed as an endocrine mediator communicating the energy needs of bone during physical activity [7, 24, 25].

The objective of this study was to examine relationships among serum osteocalcin (tOC and ucOC) and leptin with metabolic health outcomes in a large cohort of boys and girls who were at the early stages of maturation. We hypothesized that leptin and osteocalcin would be negatively related to one another, and that leptin would be a negative predictor of serum glucose, insulin and insulin resistance. In addition, we hypothesized that ucOC would be a consistent positive predictor of the various metabolic health outcomes.

## Methods

### Study design and population

This study is a cross sectional ancillary analysis of baseline data from a previously conducted double blinded placebo-controlled vitamin D supplementation trial [26] which included 318 boys and girls ages 8 to 13 years living in the United States (i.e., The GAPI Trial – University of Georgia [UGA], Purdue University [PU] and Indiana University [IU]). All study participants were healthy and absent of any chronic diseases, as determined by self- and parental report. Inclusion criteria included black and white non-Hispanic girls and boys, and sexual maturity stage 2 and 3, which was self-assessed as previously described [26]. Exclusion criteria included menarche in girls, growth disorders, or any disease, medication, or condition known to influence bone metabolism. The GAPI study was approved by the Institutional Review Boards for Human Subjects at Purdue University, the University of Georgia, and Indiana University School of Medicine. All participants and their parents or legal guardians provided informed assent and consent, respectively.

### Study variables

Blood samples were collected after 12-h fasting for the biochemical analyses of serum glucose, insulin, tOC, ucOC and leptin. Serum was stored at −80 °C until analysis. Each analyte was measured at a single laboratory for all participants.

Serum tOC and ucOC were measured in duplicate by enzyme-linked immunosorbent assay (ELISA; tOC: MicroVue™, Quidel Corp., San Diego, CA; ucOC: Takara Bio, Inc., Japan). Intra- and interassay CVs for tOC were 4.8-9.8 and 4.8-10.0%, respectively; Mean intra- and interassay CVs for ucOC were 5.2 and 8.3%, respectively. Serum ucOC measurements were only available for 170 subjects. Serum leptin and insulin were measured in duplicate by radioimmunoassay (RIA; EMD Milipore, Billerica, MA). Mean intra- and interassay CVs for insulin were 3.5 and 5.3%, respectively; and 5.0 and 4.5% for leptin, respectively. Serum glucose was measured in triplicate by microtiter modification of the enzymatic Autokit Glucose method (Wako Chemicals USA, Richmond, VA). The mean intra- and interassay CVs were 1.8 and 2.2%, respectively. The Homeostasis Model of Assessment Estimate of Insulin Resistance (HOMA-IR) was calculated as: fasting glucose (mg/dL) x fasting insulin (μU/mL)/405. The Homeostatic Model of Assessment Estimate of β Cell Function (HOMA-β) was calculated as: [360 x fasting insulin (μU/mL)]/[fasting glucose (mg/dL) – 63]. Height and weight were measured and BMI-for-age percentiles were calculated from the 2000 CDC Growth Charts [27], and obese status was defined according to percent body fat cutoffs (>25 for boys and >32% for girls) [9, 28, 29].

Percent body fat was assessed using dual-energy X-ray absorptiometry (DXA; Delphi-A, Hologic Inc [UGA]; Lunar iDXA, GE Medical Instruments [PU]; and Hologic Discovery-W [IU]). The same technician at each site conducted scans and performed analyses using instrument-specific software and protocols. Detailed procedures are described previously [26].

### Statistical analysis

Descriptive statistics were used for characterization of the study subjects. The normality of the distribution of each variable was assessed using the Shapiro-Wilk test, and variables were analyzed after logarithmic transformation. Means for quantitative variables were compared by Student’s t-tests or analysis of variance (ANOVA). Pearson correlations, partial correlations and linear regression analyses were used to determine relationships among variables with adjustments for potential confounding variables. Percent body fat was included as a covariate while examining the relationships between leptin, osteocalcin, and metabolic health outcomes. Partial correlations adjusted for percent body fat or BMI yielded similar results. Sample size for individual analyses varied according to data availability for each variable, and are described in the tables. Statistical analyses were performed using the Statistical Analysis Software, version 9.4 (Cary, NC). Statistical significance for all analyses was set at α = 0.05.

## Results

### Anthropometrics and biochemistry

Descriptive participant characteristics are presented in Table 1. The percentage of participants in sexual maturation stages 2 and 3 were 65% and 35%, respectively. Girls compared with boys were younger, shorter, and had higher percent body fat, serum leptin, and HOMA-β (all *p* < 0.001). Blacks compared with whites were younger, had higher BMI-for-age percentile, higher serum leptin, higher HOMA-β and higher serum tOC, but lower serum ucOC (all *p* < 0.050). Black females presented higher serum insulin concentrations and higher HOMA-IR, when compared with all other race by sex subgroups (both *p* < 0.050; Table 1).

**Table 1:** Anthropometric and biochemical characterization of the population according to sex and race

	Total		Black male (*n* = 82)	Black female (*n* = 80)	White male (*n* = 79)	White female (*n* = 77)	Effects		
							Sex	Race	Sex x race
	*n*	Mean (SEM)	Mean (SEM)	Mean (SEM)	Mean (SEM)	Mean (SEM)	*p*-value	*p*-value	*p*-value
Age (years)	318	11.4 (0.1)	11.8 (0.1)	10.5 (0.1)	12.1 (0.1)	11.0 (0.1)	*<0.0001*	*0.0006*	0.5396
Anthropometry									
Weight (kg)	318	47.5 (0.7)	49.4 (1.4)	48.0 (1.3)	48.0 (1.5)	44.4 (1.1)	0.1181	0.0647	0.5113
Height (m)	318	1.51 (0.01)	1.52 (0.01)	1.48 (0.01)	1.54 (0.01)	1.49 (0.01)	*<0.0001*	0.1555	0.7783
BMI-for-age (percentile)	318	68.3 (1.6)	72.1 (2.9)	77.6 (3.1)	56.9 (3.6)	66.5 (3.1)	0.1036	*0.0013*	0.3239
Body fat (%)	301	29.9 (0.6)	25.5 (1.0)	33.2 (0.9)	28.3 (1.1)	33.3 (1.0)	*<0.0001*	0.1386	0.1832
Biochemistry									
Leptin (ng/mL)	305	11.1 (0.6)	8.1 (0.9)	16.0 (1.2)	8.7 (1.2)	11.6 (0.9)	*<0.0001*	*0.0269*	0.4041
ucOC (ng/mL)	170	30.6 (1.4)	20.3 (2.4)	27.6 (2.6)	39.8 (1.7)	41.8 (3.1)	0.1415	*<0.0001*	0.1805
Total osteocalcin (ng/mL)	303	33.3 (0.7)	38.5 (1.5)	35.5 (1.6)	29.8 (0.9)	29.5 (0.8)	0.1457	*<0.0001*	0.2302
Glucose (mg/dL)	317	89.0 (0.4)	89.4 (0.8)	89.4 (0.8)	89.1 (0.8)	87.9 (0.8)	0.4352	0.2441	0.4378
Insulin (μU/mL)	318	20.3 (0.6)	18.9 (0.8)	25.9 (1.5)	18.1 (1.4)	18.0 (0.9)	*0.0006*	*<0.0001*	*0.0236*
HOMA-β	317	307.3 (13.9)	301.5 (31.8)	389.4 (36.6)	263.4 (20.7)	274.2 (13.2)	*0.0007*	*0.0015*	0.1704
HOMA-IR	317	4.5 (0.1)	4.2 (0.2)	5.7 (0.3)	4.0 (0.3)	4.0 (0.2)	*0.0020*	*<* *0.0001*	*0.0257*

Standard Error of the Mean; Body mass index; Undercarboxylated osteocalcin; Homeaostasis Model of Assessment Estimate of β Cell Function; Homeaostasis Model of Assessment Estimate of Insulin Resistance; Results of two-way ANOVA for sex, race and sex x race interactions investigated. All variables were analyzed after logarithmic transformation

The values that are italic are *p*-values indicating significance

Fifteen of the 318 total subjects (4.7%) had fasting serum glucose concentrations within the pre-diabetic range (≥100 < 125 mg/dL), with 110 mg/dL being the highest observed value. Based on percent body fat cutoffs [9, 28, 29], 51.8% (*n* = 156) of participants were classified as obese. Compared with the non-obese subjects, obese individuals had higher serum leptin, insulin, HOMA-IR and HOMA-β (all *p* < 0.0001), and lower tOC (*p* = 0.0002) (Table 2). However, ucOC concentrations did not differ between non-obese and obese groups.

**Table 2:** Biochemical characterization of the population according to nutrition status defined by percent body fat cutoffs [9, 28, 29]

		Non-obese (*n* = 145)	Obese (*n* = 156)	*p*-value
	*n*	Mean (SEM)	Mean (SEM)	
Leptin (ng/mL)	288	5.1 (0.3)	16.9 (0.8)	*<0.0001*
ucOC (ng/mL)	167	30.6 (2.1)	30.8 (1.9)	0.8644
Total osteocalcin (ng/mL)	286	36.2 (1.1)	31.3 (0.8)	*0.0002*
Glucose (mg/dL)	300	88.8 (0.6)	89.5 (0.5)	0.3608
Insulin (μU/mL)	301	17.1 (0.6)	23.6 (1.0)	*<0.0001*
HOMA-β	300	256.2 (10.8)	355.8 (25.7)	*<0.0001*
HOMA-IR	300	3.8 (0.1)	5.2 (0.2)	*<0.0001*

Standard Error of the Mean; Body mass index; Undercarboxylated osteocalcin; Homeostasis Model of Assessment Estimate of β Cell Function; Homeostasis Model of Assessment Estimate of Insulin Resistance. Results of Student’s *T*-test. All variables were analyzed after logarithmic transformation

The values that are italic are *p*-values indicating significance

### Correlations with tOC and ucOC

**Fig. 1:**
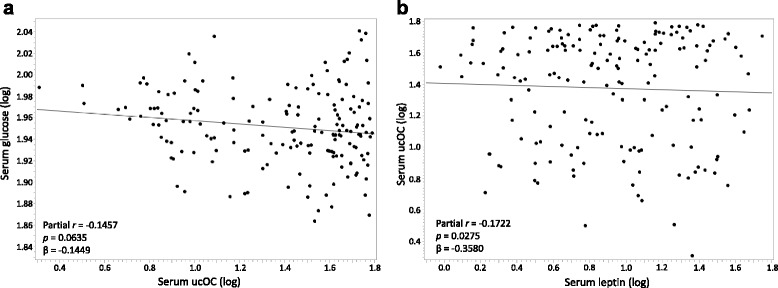
Partial regression plots between ucOC and (**a**) glucose and (**b**) leptin adjusted for age, sex and percent body fat in children living in the United States

In the unadjusted analyses, tOC was weakly negatively correlated with leptin and weakly positively correlated with height, but not correlated with any other anthropometric or biochemical outcomes (Table 3). After adjusting for percent body fat, tOC did not correlate with any of our biochemical outcomes, height or weight. Only after adjusting for percent body fat, ucOC was weakly negatively correlated with leptin and glucose. As depicted in Fig. 1, leptin remained positively, albeit weakly, associated with ucOC after adjusting for age, sex, and percent body fat.

**Table 3:** Unadjusted correlation and partial correlation adjusted for percent body fat of total osteocalcin and undercarboxylated osteocalcin with biochemical variables

		Total osteocalcin						Undercarboxylated osteocalcin				
	Unadjusted			Adjusted for percent body fat			Unadjusted			Adjusted for percent body fat		
	n	r	*p*	n	r	*p*	n	r	*p*	n	r	*p*
Leptin	303	–0.169	*0.0031*	286	0.041	0.4861	170	–0.037	0.6285	167	–0.162	*0.0368*
Insulin	303	0.001	0.9864	286	0.096	0.1057	170	–0.048	0.5345	167	–0.076	0.3282
Glucose	302	–0.026	0.6495	285	–0.033	0.5793	169	–0.150	0.0511	166	–0.159	*0.0420*
HOMA–IR	302	–0.006	0.9149	285	0.082	0.1666	169	–0.066	0.3965	166	–0.096	0.2185
HOMA–β	302	0.007	0.9060	285	0.096	0.1076	169	0.049	0.5229	166	0.036	0.6488
Weight	303	–0.008	0.8904	286	0.097	0.1028	170	–0.056	0.4672	167	–0.102	0.1923
Height	303	0.135	*0.0183*	286	0.101	0.0901	170	0.033	0.6641	167	0.035	0.6589

Homeostasis Model of Assessment Estimate of Insulin Resistance; Homeostasis Model of Assessment Estimate of β Cell Function; All variables were analyzed after logarithmic transformation

The values that are italic are *p*-values indicating significance

### Correlations with leptin

**Fig. 2:**
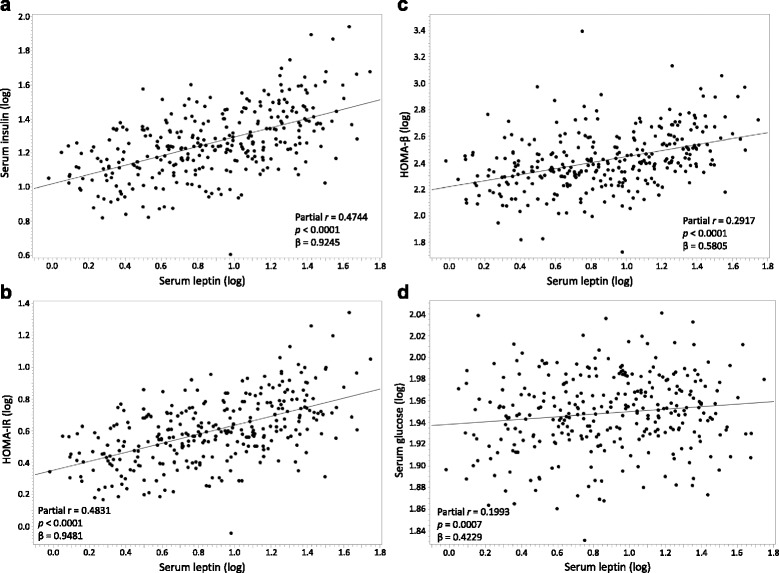
Partial regression plots between leptin and (**a**) serum insulin, (**b**) HOMA-IR, (**c**) HOMA-β, and (**d**) serum glucose adjusted for age, sex and percent body fat among children living in the United States

Leptin was strongly positively correlated with percent body fat (r = 0.878, *p* < 0.0001) and modestly positively correlated with BMI-for-age percentile (r = 0.596, *p* < 0.0001). After adjusting for age, sex, and percent body fat, leptin was a positive predictor of serum glucose (partial r = 0.20, *p* = 0.001, β = 0.4229), serum insulin (partial r = 0.47, *p* < 0.0001, β = 0.9245), HOMA-β (partial r = 0.29, *p* < 0.0001, β = 0.5805), and HOMA-IR (partial r = 0.48, *p* < 0.0001, β = 0.9481), although the strength of these associations were weak to moderate (Fig. 2).

## Discussion

Recent findings have identified new potential metabolic interactions between the skeleton, fat tissue, and energy metabolism. Whereas insulin is involved in skeletal development via the bone-forming osteoblasts [30], Karsenty & Ferron [31] have suggested that insulin, bone resorption, and osteocalcin activity are regulated by a feed forward loop, in which insulin signaling in osteoblasts decreases the expression of the gene that encodes the osteoprotegerin (*Opg*), thus increasing osteoclast-mediated bone resorption which occurs at a pH of 4.5. This acidic pH favors the decarboxylation of osteocalcin, releasing ucOC into the systemic circulation. Thereafter, ucOC plays a regulatory role in glucose metabolism by promoting pancreatic insulin secretion [32] and peripheral insulin sensitivity [3, 4, 12]. Furthermore, leptin has been shown to modulate insulin sensitivity by reducing osteocalcin bioactivity in osteoblasts [2]. Limited clinical studies have investigated the relationships between fat- and bone-derived hormonal mediators and energy metabolism [12, 18, 24, 33], and data in youth are sparse. Therefore, the primary aim of this study was to examine the relationships between osteocalcin and leptin with various metabolic health outcomes in a large cohort of early adolescent boys and girls living in the United States. Despite a weak negative correlation between leptin and ucOC, we did not find a relation between osteocalcin and insulin resistance or other metabolic health outcomes. This finding corroborates with the results from Abseyi et al. [18], in which 150 obese non-diabetic children and adolescents were evaluated and no relationship between osteocalcin and insulin resistance was found, however only tOC was assessed in that study. Another study of 248 young Finnish women also found no correlations among osteocalcin (tOC, cOC, or ucOC), glucose, insulin, and HOMA-IR [19].

Corroborating the conclusions of various other studies, data from the current study do not support the notion that osteocalcin plays a role in glucose metabolism in our cohort of children living in the United States. One key characteristic of our study participants is that they were recruited to be generally healthy and absent of any chronic disease, including prediabetes or type 2 diabetes. This is an important consideration given that the role of osteocalcin in energy metabolism may be dependent upon the presence of chronic disease. We found, however, an obesity-related difference in tOC. Takaya et al. [33] observed that adolescents with type 2 diabetes had lower ucOC levels than normal weight controls, while no difference was found between non-diabetic obese subjects and controls. In addition, differences in tOC were not observed. Another study has shown that, in overweight children only, tOC and cOC were significant negative predictors of long-term glucose control (i.e., HbA1C) [34]. Further, it is possible that osteocalcin may play a larger role in individuals with obesity-related chronic diseases such as prediabetes or type 2 diabetes. Other studies considering individuals with obesity-related chronic diseases showed similar results such that Rosato et al. [35] reported that diabetic adults had 71% lower tOC concentrations than healthy controls. Improved glycemic control after a 2-month intervention (therapy with insulin or oral medication) among diabetic subjects was associated with an increase in tOC concentrations. In addition, change in tOC was a negative predictor of fasting serum glucose concentrations in these same study participants. Meanwhile, no significant correlations were found between tOC and fasting glucose for the healthy control group. Okazaki et al. [36] also observed an increase in tOC after therapy in poorly controlled noninsulin-dependent patients.

In addition to the relatively small number of studies that have evaluated ucOC in relation to energy metabolism among adolescents, it is difficult to compare across studies because the range of values for ucOC depends on the assay employed. Applying the same method of analysis used in our study (i.e., ELISA), Van Summeren et al. [37] found mean ucOC concentrations of 31.3 ng/mL among 86 children and adolescents from 3 to 18 years of age from the Netherlands. Rochefort et al. [38] observed mean ucOC of 34.5 ng/mL among 13 nonobese children and 34.3 ng/mL among 27 obese children aged 9 to 12 years old. Using RIA, Pollock et al. [12] found mean ucOC of 5.6 ng/mL among normal glucose subjects and 7.8 ng/mL among subjects with prediabetes in a group of U.S. overweight children from 7 to 11 years old. Tubic et al. [34], in a study with 108 Swedish 2–9 year old children, found mean tOC and ucOC of 82.6 ng/mL and 7.0 ng/mL, respectively, among normal weight subjects and mean of 77.0 ng/mL and 7.9 ng/mL among overweight subjects, however differences were not significant. Alfadda et al. [39] found median levels of 0.53 ng/mL for cOC, 1.14 ng/mL for ucOC and 8.8 ng/mL for tOC in a group of adults with type 2 diabetes, and they were lower among overweight/obese adults in comparison to what has been reported with normal individuals. In their study, however, tOC was unexpectedly higher than the value obtained by simply summing the two individual fractions. While tOC was measured by ELISA, Alfadda et al. [39] measured cOC and ucOC fractions by enzyme immunoassay (EIA). For these reasons, it is important to take note of the differences among the methods established for measuring osteocalcin, considering that values derived from the RIA appear consistently lower than those from ELISA, the latter of which we used in the current study.

In our study, leptin and ucOC were negatively correlated independent of percent body fat. Leptin, in turn, was a strong positive correlate of insulin resistance and β-cell function. Despite the effect of leptin on reducing insulin secretion [40], the positive correlation of leptin with HOMA-β (an estimate of pancreatic β-cell function) was expected. In non-diabetic populations – especially among youth – increased insulin secretion precedes insulin resistance [41] and, in the presence of obesity, indicates a risk of developing type-2 diabetes [42]. In contrast to earlier pediatric data, neither tOC nor ucOC correlated with HOMA-β. Pollock et al. [12] showed positive associations between ucOC and multiple markers of β-cell responsiveness. However, these findings were evident only in children with pre-diabetes and not those with normal glucose control. The participants in the current study were healthy and absent of any chronic diseases, thus the effect of osteocalcin in modulating insulin secretion may be confined to individuals at risk of developing type-2 diabetes. These data were later supported by Gower et al. [43], but rather in a cohort of overweight and obese adults (mean age = 34.9 ± 8.3 years).

Sex-related differences in energy metabolism have been well characterized, with girls being more insulin resistant versus their male counterparts [44–46]. Sex steroids and genetics may partially explain why girls are more inclined than boys to develop disturbances in glucose metabolism [46]. However, as shown by Jeffery et al. [47], the greater adiposity in girls versus boys is also a contributor. In our study, the higher percent body fat in girls was accompanied by higher HOMA-β with HOMA-IR being greatest in black girls. Lee et al. [45] reported that female subjects had significantly higher mean HOMA-IR than male subjects (*p* = 0.02). In addition, overweight and obese adolescents had higher HOMA-IR levels compared with normal weight adolescents [45], and no other factor was more influential on HOMA-IR than obesity status. Consistent with these findings, the overweight/obese versus normal weight children in the current study had higher HOMA-IR values, likely due to corresponding elevations in fasting serum insulin in the overweight/obese individuals.

Racial differences in markers of insulin resistance and sensitivity have been found by others [44, 48], but not consistently in all studies [45]. We observed that black subjects had higher HOMA-β, BMI-for-age percentile, serum leptin and tOC than white subjects, and lower ucOC. Black girls also had higher insulin, and consequently HOMA-IR, than the other race by sex groups. A meta-analysis with 74 study cohorts including 3,813 individuals showed that Africans had significantly lower insulin sensitivity and higher insulin response than Caucasian and East Asian subpopulations [48]. This study also found that the stabilization points in the hyperbolic relationship between insulin sensitivity and insulin response in Africans with normal glucose tolerance were located around unstable extreme points in the curve, where a small change in one variable is associated with a large nonlinear change in the other variable. Therefore, among Africans, even a small increase in insulin resistance could lead to a rapid increase in the amount of released insulin required to maintain normal glucose tolerance, which suggests that this particular subpopulation is more vulnerable to the development of type-2 diabetes. Indeed, the prevalence of type-2 diabetes is 13.2% in people of African descent versus 7.6% in people of non-Hispanic European decent [49].

Our study has some limitations. Because of the cross-sectional design, these results do not allow causal inference or temporal associations. Unfortunately, we were unable to collect data on dynamic measures of peripheral glucose metabolism or pancreatic β-cell function. However, compared to an oral glucose tolerance test, HOMA-IR performed well as a measure of glucose metabolism in obese children and adolescents [50] and has been used previously in pediatric populations of similar age and stage of sexual maturation of the children included in the present study [51]. Our study included healthy children in a narrow range of puberty, thus generalizability to children at all stages of maturation is limited. Additionally, our subjects represented otherwise healthy children and included only a small number (i.e. < 5%) who presented with a fasting serum glucose within the pre-diabetic range.

## Conclusions

This ancillary cross-sectional analysis shows that leptin concentrations negatively correlated with ucOC and positively correlated with insulin resistance and β-cell function estimates in healthy children. However, associations between OC and markers of energy metabolism were not observed. Whereas our findings do not support the position that OC plays a direct role in energy metabolism in healthy children, these relationships warrant additional attention within the context of obesity and/or obesity-related chronic disease specifically through prospective study designs throughout adolescence.

## Abbreviations

ANOVA: Analysis of variance
BMI: Body mass index
DXA: Dual-energy X-ray absorptiometry
cOC: Carboxylated osteocalcin
ELISA: Enzyme-linked immunosorbant assay
HOMA-IR: Homeostasis Model of Assessment Estimate of Insulin Resistance
HOMA-β: Homeostatic Model of Assessment Estimate of β Cell Function
LEPR: Leptin receptor
OPG: Osteoprotegerin
RANKL: Receptor activator of nuclear factor κB ligand
RIA: Radioimmunoassay
tOC: Total osteocalcin
ucOC: Undercarboxylated osteocalcin
